# Lactic acid bacteria—20 years exploring their potential as live vectors for mucosal vaccination

**DOI:** 10.1007/s00253-015-6498-0

**Published:** 2015-03-10

**Authors:** Agnieszka Wyszyńska, Patrycja Kobierecka, Jacek Bardowski, Elżbieta Katarzyna Jagusztyn-Krynicka

**Affiliations:** 1 Department of Bacterial Genetics, Institute of Microbiology, Faculty of Biology, University of Warsaw, Miecznikowa 1, 02-096 Warsaw, Poland; 2 Institute of Biochemistry and Biophysics, Polish Academy of Sciences, Pawinskiego 5a, 02-106 Warsaw, Poland

**Keywords:** Lactic acid bacteria, Antigens, DNA vaccine, GEM particles, Immunoprophylaxis

## Abstract

Lactic acid bacteria (LAB) are a diverse group of Gram-positive, nonsporulating, low G + C content bacteria. Many of them have been given generally regarded as safe status. Over the past two decades, intensive genetic and molecular research carried out on LAB, mainly *Lactococcus lactis* and some species of the *Lactobacillus* genus, has revealed new, potential biomedical LAB applications, including the use of LAB as adjuvants, immunostimulators, or therapeutic drug delivery systems, or as factories to produce therapeutic molecules. LAB enable immunization via the mucosal route, which increases effectiveness against pathogens that use the mucosa as the major route of entry into the human body. In this review, we concentrate on the encouraging application of *Lactococcus* and *Lactobacillus* genera for the development of live mucosal vaccines. First, we present the progress that has recently been made in the field of developing tools for LAB genetic manipulations, which has resulted in the successful expression of many bacterial, parasitic, and viral antigens in LAB strains. Next, we discuss the factors influencing the efficacy of the constructed vaccine prototypes that have been tested in various animal models. Apart from the research focused on an application of live LABs as carriers of foreign antigens, a lot of work has been recently done on the potential usage of nonliving, nonrecombinant *L. lactis* designated as Gram-positive enhancer matrix (GEM), as a delivery system for mucosal vaccination. The advantages and disadvantages of both strategies are also presented.

## Characteristics of lactic acid bacteria

Lactic acid bacteria (LAB) are Gram-positive, nonsporulating bacteria, with low-GC content genomes. This group is distinguished by the ability to carry out fermentation of carbohydrates to form lactic acid. This group of microorganisms includes cocci and bacilli, representatives of the species belonging to the genera of *Lactococcus*, *Lactobacillus*, *Streptococcus*, *Pediococcus*, *Leuconostoc*, *Bifidobacterium*, and several others. Many LAB have received generally regarded as safe (GRAS) status from the American Food and Drug Administration (FDA), but it is worthwhile to mention that pathogenic bacteria such as *Streptococcus pyogenes* and *Streptococcus pneumonia* also belong to this group (Daniel et al. 2011). Bacteria from the LAB group are characterized by the absence of genes in their genomes that code for proteins involved in various biosynthesis pathways. Accordingly, these auxotrophic organisms occur in environments rich in amino acids, purines, and pyrimidines, where their high nutritional requirements can be met.

Until recently, bacteria from the LAB group have mainly been used in the production and preservation of foods. Some species are recognized as probiotics, which—according to the definition proposed by the World Health Organization (WHO)—confer a health benefit on the host when administered in adequate amounts (de Vos 2011; FAO/WHO 2001). However, LAB strains recognized as probiotics differ in their activities. Some bacteria of this group modulate the composition of the bacterial flora of the intestine and thereby maintain homeostasis of the intestinal microbiota. In addition, several of probiotics strains stimulate the immune system and reduce the risk of allergic reactions. Other bacterial species provide protection against pathogenic bacteria by competing with them for the colonized surface, by producing compounds that inhibit the growth of pathogens, or by inducing the production of mucus and antimicrobial peptides (AMP) by the mucosal epithelial cells (Isolauri et al. 2004). Probiotic strains are of utmost importance in supporting the treatment of diseases of the digestive system (mainly diarrhea of viral etiology or diarrhea associated with the use of antibiotics), inflammatory bowel disease (IBD), and autoimmune disorders (Fontana et al. 2013; Isolauri et al. 2004).

For more than 20 years, LAB have been intensively studied as potential bacterial carriers of compounds with therapeutic or prophylactic effects (Bermudez-Humaran et al. 2011). LAB enable immunization via the mucosal route, which is not only simpler than the standard injections but also increases the effectiveness against pathogens that use the mucosa as their major route of entry into the human body. Delivery of antigens using LAB strains may induce both mucosal (sIgA) and systemic immune responses (Bermudez-Humaran et al. 2011). The attractiveness of LAB in immunoprophylaxis and therapy is also determined by their resistance to the low pH of gastric juice and, for certain strains, the ability to adhere to the surface of the intestinal epithelium. Moreover, some LAB strains have adjuvant properties, which means that they can enhance the immune response induced by the carried antigen. Thanks to the possibility of lyophilization, LAB do not require storage at low temperature, and administration of the preparation does not require specialized personnel.

All of these characteristics, in particular, the health-promoting properties and a high degree of safety of LAB, make them an attractive alternative to other vectors used for the construction of vaccines, including attenuated strains of various species of pathogenic microorganisms, liposomes, and microparticles. This review examines the progress in using LAB for immunoprophylaxis.

## Genetic engineering tools used for cloning heterologous genes in *Lactococcus lactis*

So far, *L. lactis* remains the model microorganism in LAB research. Rapid research progress on the use of LAB for treatment and prophylaxis occurred at the turn of the twenty-first century. In these studies, plasmid-cured strains of *L. lactis* subsp. *lactis* IL1403 and *L. lactis* subsp. *cremoris* MG1363, whose genomes were sequenced in 1999/2001 and 2007, respectively, are most commonly used (Bolotin et al. 2001; Wegmann et al. 2007; Linares et al. 2010). Currently, databases provide genetic information on 33 strains of the species *L. lactis* (http://www.ncbi.nlm.nih.gov/genome/genomes/156, November 2014). The first vectors for cloning foreign genes in LAB were developed about 30 years ago, on the basis of the replication systems of the two cryptic, broad host-range plasmids of *L. lactis*: pWV01 and pSH71 (de Vos 1987; Kok et al. 1984). To date, many derivatives of these plasmids have been created (Shareck et al. 2004). Another widely used replicon is pAMbeta-1, isolated from *Enterococcus faecalis*. On the basis of its replication system, the low-copy plasmid pIL252 and the high-copy plasmid pIL253 were constructed (Simon and Chopin 1988; Shareck et al. 2004). A number of different systems for the expression of genes encoding heterologous proteins in the cells of *L. lactis* were developed using both constitutive and inducible promoters. The most commonly used system for expression of heterologous proteins is the NICE system, utilizing the nisin promoter (Mierau and Kleerebezem 2005). Many of the developed expression systems have inducible promoters whose expression depends on environmental conditions. These include, for example, the P_170_ promoter, active at low pH and subject to self-induction by accumulated lactic acid when the culture enters the stationary phase of growth (Madsen et al. 1999), or the *zit* operon promoter that is regulated by ZitR protein and is activated by low concentration of zinc ions (the so-called *zinc hunger*) (Llull and Poquet 2004). The recently described Zirex system enables induction of genes regulated by the concentration of zinc ions and by the use of the pneumococci regulatory protein SczA (Mu et al. 2013). In studies aimed at examining the potential application of LAB in immunoprophylaxis, promoters induced under conditions prevailing in the immunized body are particularly useful. One such expression system is stress inducible controlled expression system (SICE). The SICE system uses a promoter of the *L. lactis groESL* operon, whose expression is induced under stress conditions (Benbouziane et al. 2013).

## Genetic engineering tools used for cloning heterologous genes in *Lactobacillus* spp.

In recent years, interest in bacteria of the genus *Lactobacillus* has also increased. More than 180 species are included in this genus (http://www.ncbi.nlm.nih.gov/Taxonomy/Browser/wwwtax.cgi?id=1578, August 2014). However, most attention is focused on the strains with proven probiotic properties; among these are the following bacterial species: *Lactobacillus rhamnosus*, *Lactobacillus casei, Lactobacillus bulgaricus*, *Lactobacillus salivarius*, *Lactobacillus plantarum*, *Lactobacillus acidophilus*, *Lactobacillus helveticus*, and *Lactobacillus gasseri* (Fontana et al. 2013).

Preparation of expression vectors for gene cloning in cells of the genus *Lactobacillus* is challenging, mainly due to the unusually high level of genetic diversity. Some of the plasmid replication systems described are only active in specific strains, and known lactobacilli promoters have different activity levels, depending on the strain they are in. The most commonly used cloning vectors for *L. plantarum*, *L. acidophilus*, and *L. gasseri* are those having the replication system of pWV01, pSH71, or pAMbeta-1 (Stoeker et al. 2011; Bron et al. 2004b; Duong et al. 2011; Kajikawa et al. 2011, 2012).

In the expression vectors for *Lactobacillus*, both constitutive promoters—such as P_pgm_ (phosphoglycerate mutase promoter), P_ldhl_ (lactate dehydrogenase promoter), or P_slpA_ (promoter of the gene encoding the S-layer of the protein SlpA)—and inducible promoters are used. One of them is the promoter of a gene coding for a heat shock protein from *Enterococcus faecium*; its activity has been analyzed in cells of *L. plantarum* (Maidin et al. 2014). Recent global analysis of lactobacilli transcriptomes, or the investigation of genome libraries using various reporter genes, has resulted in identification of promoters induced by specific environmental conditions. An analysis of the transcriptome of *L. acidophilus* identified promoters that are induced by the presence of carbohydrates, i.e., P_FOS_ (fructo-oligosaccharide), P_lac_ (lactose), and P_tre_ (trehalose), but are repressed in the presence of glucose. P_FOS_ may prove to be particularly useful in therapy or immunoprophylaxis, as fructo-oligosaccharides are prebiotics that stimulate the development of the intestinal microflora (Duong et al. 2011). Using a gene encoding alanine racemase as a promoter probe for the genome-wide identification of inducible *L. plantarum* genes resulted in identification of many genes whose expression is induced by high salt concentration or by bile salts (Bron et al. 2004a, c).

## LAB as carriers of heterologous bacterial, parasitic, and viral antigens

LAB are characterized by their high physiological and genetic diversity. Thus, the abilities of different strains to persist and multiply in an immunized organism differ substantially. Moreover, dissimilarities in the composition of their cell walls results in significant differences in the stimulated immune response. Some cell-wall components such as peptidoglycan, lipoproteins, or lipoteichoic acids are recognized by eukaryotic Toll-like (TLR) or nucleotide oligomerization domain (NOD) receptors involved in the anti-inflammatory immune response. Strain-specific effects of LAB result from the induction of diverse immune regulatory pathways (Zeuthen et al. 2008; Macho Fernandez et al. 2011). Therefore, the immunization schedule developed for vaccines that have been generated on the basis of one member of the LAB group as an antigen carrier is often unsuitable for immunization of different host targets. The data obtained by different research groups are not consistent, and the difficulties comparing them result from the use of different antigens, vectors, and immunization schedules. Parameters affecting the immune response induced by prototypes of LAB vaccines will be discussed in a later part of this subsection.

### Routes of administration

The first use of LAB as a vaccine vector is a 1990 report of using formalin-killed *Streptococcus lactis* cells that produced PAc protein (antigen I/II) on the cell surface to immunize against *Streptococcus mutans*. Intragastric immunization of mice resulted in the production of specific IgG and IgA antibodies; thus, it was shown, for the first time, that LAB could be an attractive alternative to conventional bacterial carriers of foreign antigens (Iwaki et al. 1990).

Most of the prototype vaccines using LAB strains as carriers addressed infectious diseases caused by human pathogens that penetrate through the mucous membranes of the gastrointestinal, respiratory, and urogenital tracts. For this reason, different routes of administration were examined. For example, in the case of *S. pneumoniae*, the efficacy of intranasally administered vaccines was evaluated (Hanniffy et al. 2007), whereas in the case of *Helicobacter pylori*, oral or intragastric vaccines were the first to be analyzed (Li et al. 2014). Because the *mucosa-associated lymphoid tissue* (MALT) mucous-membrane immune system constitutes one network covering the whole body, and because lymphocytes are able to migrate, oral immunization also provides systemic immunity expressed by the mucous membranes of other organs. LAB strains, acting as carriers of *S. pneumoniae* antigens, have already been shown to be effective at intranasal immunization (see below). Thus, based on actual knowledge about MALT functioning, an *L. lactis* strain, comprising the *pppA* gene of *S. pneumoniae* expressed from a nisin-inducible promoter, was also employed for oral immunization of adult, as well as young, mice. Both the induction of specific antibodies in the gut and the stimulation of a systemic immune response (IgG in the serum) were observed. This immunization raised the resistance of mice to infection, although the effect was dependent on the serotype of the strain used in the protective experiment (Villena et al. 2008, 2010).

### Carrier strains

Many studies on the effectiveness of various representatives of the LAB group as carriers of heterologous antigens for vaccination were conducted with the highly immunogenic tetanus toxin C-terminal fragment (TTFC) as antigen. This protein fragment has been intensively studied as a replacement for inactivated toxin in the combined diphtheria, tetanus, and pertussis (DTP) vaccine. The antigen was produced in the cells of several species of bacteria from the LAB group (*L. lactis*, *Lactobacillus* spp., and *Streptococcus gordonii*) that differ in their ability to survive in colonized ecological niches. Studies with *L. lactis* and *L. plantarum* as TTFC carriers indicated that the immunogenicity of *L. lactis* was significantly lower than that of *L. plantarum*, particularly when those strains had a mutation in the *alr* gene encoding alanine racemase, an enzyme involved in cell-wall synthesis. Intestinal TTFC-specific immunoglobulin A was induced only after immunization with the recombinant *L. plantarum* mutant strain. This conclusion, however, was not unequivocal because the gene encoding TTFC was expressed in the LAB carriers from promoters that are difficult to compare (a nisin-inducible promoter and a constitutive promoter) (Grangette et al. 2004).

The effect of the carrier on the level of immune response was also analyzed with respect to the efficacy of a potential antimalaria vaccine. Strains producing the well-characterized merozoite surface antigen (MSA2), a surface protein of *Plasmodium falciparum*, were tested, using a number of variants. The antigen produced in the cells of *L. lactis*, *L. reuteri*, and *L. salivarius* was associated with peptidoglycan of the live cells by either covalent or noncovalent binding, or was located on the surface of cells treated with trichloroacetic acid (TCA). In addition, genetically different mouse lines were used in the studies. The combined nasal and oral immunization was employed in this work. Significant differences in the levels of induction and the types of induced immune response were observed, depending on the type of animal immunized, the genus of the carrier, and the location of the antigen (Moorthy and Ramasamy 2007). *L. lactis* and *L. plantarum* (*alr* ^*−*^ *)* strains expressing the *H. pylori* B-subunit of urease (UreB) were also analyzed for anti-*Helicobacter* immunization. A positive effect (induction of the specific antibodies and reduction of the level of colonization of mice by *H. felis*) was only seen after vaccination with a *Lactobacillus* strain (Lee et al. 2001; Corthesy et al. 2005). It is postulated that some *L. lactis* strains that reveal high affinity to mucus are promising candidates for development of a vaccine against bird flu (Szatraj et al. 2014; Radziwill-Bienkowska et al. 2014).

The impact of the carrier on induction of the immune system and the effectiveness of the vaccine prototype were also analyzed in relation to vaccines against *S. pneumoniae*. Tests were mainly carried out in a mouse model after intranasal immunization. The licensed anti-*S. pneumoniae* vaccines contain bacterial polysaccharides [*polysaccharide vaccine* (PSV)], which in some preparations are conjugated to a carrier protein (CMR197—*inactive mutated diphtheria toxin*). Strains of *S. pneumoniae* are characterized by a high diversity of serotypes (more than 90 serotypes have been described so far), and so vaccines are effective only against those serotypes whose polysaccharides are included in the formulation. Due to the multidrug resistance of the *S. pneumoniae* pathogen and the different geographic distributions of its serotypes, many studies are being conducted to develop effective formulations based on conserved protein antigens. Several protein antigens [*Pneumococcal surface antigen* (PsaA), *Pneumococcal surface protein* (PspA), and *Pneumococcal protective protein* (PppA)] produced by *Lactobacillus* and *Lactococcus* species at various cellular locations have been examined as protective antigens. Extensive information on the potential use of LAB for anti-*S. pneumoniae* immunization can be found in the excellent review by Villena et al. (2011). Preliminary experiments proved that intranasal administration of *L. lactis* that produced the intracellular antigen PspA to mice was more effective than intranasal or subcutaneous immunization with the purified recombinant protein (Hanniffy et al. 2007). The first immunization attempts using an *L. casei* strain that contained the *pspA* gene of *S. pneumoniae* cloned on a plasmid and under the control of an inducible lactose promoter showed no stimulation of the immune response, probably due to low levels of the antigen. Therefore, for the comparison of the immunogenicity of the LAB strains carrying the *pspA* gene, a strong constitutive promoter was employed. Among the four species tested (*L. lactis*, *L. casei*, *L. plantarum*, and *L. helveticus*), used for nasal mice immunization, the lowest level of immune response was seen for *L. lactis*, which correlated with a low level of produced antigen and a short nasal mucosa residence time for the carrier strain. Strains of the *Lactobacillus* genus colonized the nasal mucosa for about 3 days and stimulated a significant level of both IgG and IgA. The studies also indicated differences in the level of induced immune response between various species of *Lactobacillus* genus. The authors suggested that it reflects differences in their intrinsic adjuvant potential (Oliveira et al. 2006). The adjuvant effect of *L. plantarum* was also observed in analysis of the effectiveness of two types of LAB, carriers of the HPV E7 antigen, as therapeutic cancer vaccines. Although *L. plantarum* induced a lesser immune response than *L. lactis*, the *L. plantarum* vaccination resulted in a much more rapid regression of tumors (Cortes-Perez et al. 2007).

### The roles of antigen location and amount

The influence of antigen location, inside or on the surface of carrier-strain cells, on the efficacy of vaccination has been tested multiple times. Location—along with the route of administration, the immunization schedule, the genus/species of the carrier strain, and the amount of antigen—is certainly one of the factors that determines the level and the type of induced immune response in the host. Research so far yields no clear answer about the most effective cellular location of the antigen for immunization.

Foreign proteins in the cytoplasm of LAB, even if they are secreted by the natural host (e.g., tetanus toxin C-fragment), typically trigger a satisfactory level of immune response (humoral and cellular) (Grangette et al. 2002; Reveneau et al. 2002). Such localization avoids degradation of antigens by proteases present in the gastrointestinal tract, and it also makes them available to the immune system of the host only after lysis of the carrier strain cells. The release of larger quantities of cytoplasmic antigen in vivo can be ensured using strains with a knockout *alr* gene encoding alanine racemase (Grangette et al. 2004; Hugentobler et al. 2012a; Corthesy et al. 2005). The production of large quantities of foreign protein in the cytoplasm is sometimes lethal to the carrier cells, or the protein may be intensively degraded. The use of strains that do not produce exo- or intracellular proteases such as HtrA and Clp may sometimes allow appropriate production of heterologous antigen (Morello et al. 2008). In some cases, the only effective strategy is to change the location of the antigen. Intracellular production of the E7 protein of human papillomavirus type 16 (HPV-16) resulted in its rapid degradation. The situation was not improved in an *L. lactis* strain incapable of producing ClpP protease. Larger quantities of E7 were only obtained by anchoring of the E7 antigen to the cell wall or releasing it into the environment (Bermudez-Humaran et al. 2002; Cortes-Perez et al. 2003). Several strategies to “send” heterologous proteins to the cell surface of LAB strains or into the environment have been developed. For antigen secretion, the signal sequence of the protein Usp45 (*unknown secreted protein of 45 kDa*)—the only *L. lactis* protein secreted in significant quantities by *L. lactis*—is generally used (van Asseldonk et al. 1990). The Usp45 signal sequence also led to satisfactory secretion in *Lactobacillus* species. The method to increase the efficiency of secretion is to fuse a nucleotide sequence encoding the antigenic protein to a nucleotide sequence encoding the synthetic peptide LEISSTCDA (Guimaraes et al. 2006). Insertion of this peptide potentially affects precursor conformation and thus facilitates its processing by cytoplasmic secretory chaperones, or it might optimize the charge balance around the signal cleavage site to facilitate translocation (Le Loir et al. 1998).

Two strategies are used to present heterologous antigens on the surface of LAB. The first makes use of the C terminus of cell-anchored proteins that contain the LPXTG recognition motif. Covalent cell-wall attachment of fusion proteins carrying this motif depends on the activity of sortase A (SrtA), an enzyme with transpeptidase activity (Call and Klaenhammer 2013). To date, the LPXTG motifs of the PrtP proteinase of *Lactococcus* (Ramasamy et al. 2006), the PrtB proteinase of *L. delbrueckii* subsp. *bulgaricus* (Kim et al. 2008), the M6 protein of *S. pyogenes* (Dieye et al. 2001; Ribeiro et al. 2002; Wieczorek and Martin 2010), and protein A of *Staphylococcus aureus* have been used to ensure surface localization of different antigens (Steidler et al. 1998b). The second approach is based on the peptidoglycan-binding (PA) domain of some lactococcal proteins, such as AcmA, the major autolysin of *L. lactis* (Buist et al. 1997). The PA is located in the C terminus of AcmA and comprises three LysM motifs, each with about 45 amino acids, separated by spacer sequences. After secretion, AcmA is directed to the cell wall and its C terminus determines its noncovalent binding to the cell-wall peptidoglycan (Buist et al. 2008). It has been demonstrated that hybrid PA fusions exhibited similar binding (Bosma et al. 2006; Steen et al. 2003; Raha et al. 2005). In addition, the LysM domains of other Gram-positive bacteria bind heterologous proteins to the cell-wall peptidoglycan (Xu et al. 2011; Turner et al. 2004; Hu et al. 2010). In 2011, lipoprotein basic membrane protein A (BmpA), a component of the classic ABC transporter responsible for the import of purines, was identified (Berlec et al. 2011). BmpA is covalently anchored to the membrane bilayer. The possibility of using this protein as a carrier of antigens is currently being examined (Zadravec et al. 2014).

Determination of the genomic sequence of many LAB strains has enabled analysis of their proteomes, including the secretome (the set of proteins forming the cell envelope and those secreted into the environment). These data may be used to search for new strategies aimed at manipulating the localization of foreign antigens in the cells of the carrier.

The first study aimed at determining the significance of antigen location for immune response induction used the above-mentioned tetanus toxin C-fragment (TTFC) (Reveneau et al. 2002). In that work, the authors evaluated the immunogenicity of recombinant strains of *L. plantarum* producing variants of TTFC that went to the cytoplasm, to the cell wall or to the environment. They also tested three routes of immunization in mice: subcutaneous, intragastric, or intranasal. The immune responses (specific IgG in serum) established after subcutaneous application did not depend on the location of the antigen. In contrast, when administered to mucosal surfaces of the stomach or nose, the best results were obtained when the antigen was present in the cytoplasm. Cytoplasmic production led to the highest production of TTFC and that was believed to be the cause for the differences observed. The quantity of antigen in the carrier cells after intragastric administration is very important. A significant immune response was observed following administration of *L. plantarum* containing high amounts of TTFC protein (Grangette et al. 2002). When lower amounts of the antigen were present in the cytoplasm, the levels of IgG antibodies only increased after intranasal administration. Furthermore, these antibodies did not neutralize the tetanus toxin. A protective effect was only observed when LAB strains producing high concentrations of the antigen were administered to the nasal mucosa, which suggests that the amount of the antigen is important for inducing antibodies with a high avidity (Grangette et al. 2001, 2002).

The use of different carrier strains or different routes of administration often makes it impossible to compare and evaluate the significance of antigen localization in the induction of an immune response. For this reason, the studies of Marelli et al. on the use of *L. lactis* as the carrier of the rotaviral VP8 protein are noteworthy. In mice that were orally immunized with a *L. lactis* strain producing the cytoplasmic form of VP8 antigen, significant levels of intestinal IgA antibodies were observed. But when the VP8 antigen was anchored to the *L. lactis* cell wall by *S. pyogens* M6 protein cell wall anchor domain, the immune response included both specific sIgA antibodies throughout the mucus membranes of the gastrointestinal tract, as well as induction of IgG antibodies. Subsequently, the infectious ability of viral particles incubated with antibodies isolated from the immunized animals was studied using the MA-104 cell line (fetal monkey kidney cell line). Antibodies from animals that were immunized using the *L. lactis* strain with VP8 protein localized in the envelope showed 100 % neutralization of the viral particles, whereas antibodies from animals that were immunized using a strain with cytoplasmic localization of the antigen showed only 50 % reduction of viral infectivity (Marelli et al. 2011). The use of VP7 protein, a different rotavirus antigen, provided contradictory data. Antibodies induced by a form of the protein anchored in the cell envelope did not neutralize viral particles. The highest immunogenicity was exhibited by the *L. lactis* strain that secreted VP7 protein into the environment (Perez et al. 2005).

It has also been observed that the type of anchoring motif on the surface of the antigen-presenting LAB cells may significantly affect the efficacy of vaccination (Kajikawa et al. 2011). In the recent literature, the importance of the fate of the antigen after it is delivered to the immunized organism is also discussed. For example, an extremely positive effect was obtained by generating a fusion of the protective antigen (PA) of *Bacillus anthracis* with a peptide that delivers it to dendritic cells (DCs) (Mohamadzadeh et al. 2009, 2010). Enhancement of the immune response was also obtained by coexpression of a *Salmonella enterica* gene encoding flagellin, a protein-stimulating TLR5 receptors, and a gene encoding the Gag HIV-1 protein in an *L. acidophilus* strain (Kajikawa et al. 2012).

### Comparison of the effectiveness of live LAB strains and GEM particles

In 2005, a new carrier system was developed using nongenetically modified LAB (Bosma et al. 2006). When LAB cells are treated with hot trichloroacetic acid, which deprives them of surface lipoteichoic acids, proteins, and the cytoplasmic content, the peptidoglycan remains intact and provides a particle shape similar to live cells. The resulting particles are known as Gram-positive enhancer matrix (GEMs). The procedure applies the antigen–PA fusion proteins produced and purified from (recombinant) *Escherichia coli* or *L. lactis*, which subsequently are mixed with GEM particles. GEM particles reveal higher binding ability of fused proteins containing PA domain than live LAB cells. Thus, this strategy offers an opportunity to use nongenetically modified organism (non-GMO) and ready-to-go formulation. The lack of recombinant DNA in this procedure eliminates the risk of uncontrolled dissemination of nucleic acids into the environment. An additional advantage of this strategy is the stability of GEM particles and the possibility of long-term storage (Bosma et al. 2006; van Roosmalen et al. 2006). Additionally, it has been recently shown that the simple mixing the subunit vaccine with the GEM particles results in a strongly enhanced immune response especially after intranasal application (see below). Thus, the GEM particles produced from a bacterium, which is used in the production of dairy products, are more safe in comparison to other adjuvants and can be considered as a candidate adjuvant for mucosal use in humans. However, GEM particles have one disadvantage compared to using live LAB strains (*Lactobacillus*) for immunization. Live strains can colonize the gut, reducing the number of vaccine doses needed and significantly simplifying the immunization procedure.

So far, GEM particles have been tested as carriers to immunize against three pathogens: *S. pneumoniae* [IgA1 protease (IgA1p), putative proteinase maturation protein A (PpmA), and streptococcal lipoprotein (SlrA)], *Yersinia pestis* (the LcrV protein), and *P. falciparum* (the merozoite surface antigen MSA2) (Audouy et al. 2006, 2007; Ramasamy et al. 2006; Ramirez et al. 2010). The immunogenicity and protective efficacy of *L. lactis* GEM particles displaying *Y. pestis* LcrV was investigated in a neonatal mouse model. Newborn mice immunized intranasally with GEM-LcrV developed LcrV-specific antibodies, Th1-type cell mediated immunity, and were protected against lethal *Y. pestis* (plague) infection (Ramirez et al. 2010). GEM presenting MSA2 used to oral immunizations of rabbits was equally efficient in eliciting antigen-specific antibody responses as live *L. lactis* cells producing the same antigen (Ramasamy et al. 2006). Intranasal immunization with GEM particles displaying three pneumococcal antigens (PpmA, SlrA, and IgA1p) showed significant protection against fatal pneumococcal pneumonia in mice (Audouy et al. 2006, 2007). It was also demonstrated that the GEM particles may constitute a platform for presentation of several antigens at the same time, which may be significant for immunization against pathogens with a high genetic variability, such as *Campylobacter jejuni*, a leading cause of food-borne gastrointestinal diseases (Kobierecka et al. 2015; Wyszynska et al. 2004). The results illustrate the potential of using nongenetically modified *L. lactis* as a safe vaccine delivery vehicle to elicit systemic antibodies, thereby avoiding the dissemination of recombinant DNA into the environment. When using GEM particles as a platform for presenting of heterologous antigens, intranasal immunization is generally most effective in respect to the induced immune response as well as to the protective effect.

GEM particles, though devoid of surface proteins, retain inflammatory properties characteristic of live bacteria (Audouy et al. 2006, 2007) and have adjuvant properties. While analyzing the adjuvant properties of GEM particles, a commercially available, monovalent H3N2 vaccine, containing hemagglutinin and GEM particles, was administered to mice intranasally. In contrast to other experiments, the influenza virus antigen was not anchored on the surface of the GEM particles, but rather constituted a separate component of the vaccine. While the resulting systemic response (IgG) was similar to mice vaccinated intramuscularly with influenza vaccine, the mucosal response (sIgA) was significantly higher for the intranasal vaccination with GEM particles. An increased number of cells producing IFN-γ was also observed, which indicates the dominant response of the Th1-type lymphocytes (Saluja et al. 2010a). When GEM particles are used as an adjuvant, the hemagglutinin dose can be reduced up to fivefold and still induce a protective effect in mice (Saluja et al. 2010b).

These promising results attracted interest from Mucosis B.V., a Dutch biotechnology company (mucosis is the first spin-off of the Biomade Technology Foundation) (Audouy et al. 2006). A phase I clinical trial of a mucosis influenza vaccine for human use (FluGEM) is almost finished. FluGEM is the only vaccine, based on LAB as carriers, to reach this stage of investigation.

## LAB as DNA vaccines

Genetic immunization with plasmid DNA has been studied for over 20 years, for both immunoprophylaxis of infectious diseases and also cancer immunotherapy. DNA immunization leads to the induction of both humoral and cellular immune responses. Plasmid DNA can be delivered by intramuscular injection (the so-called naked DNA immunization) or using suitable bacterial carriers. The intensive studies conducted so far have focused on the use of attenuated, enteroinvasive bacteria, such as *Salmonella* spp., *Yersinia enterocolitica*, or *Listeria monocytogenes*, as well as appropriately modified *E. coli* cells producing O listeriolysine (*L. monocytogenes*) and/or invasin (*Y. enterocolitica*) as DNA carriers (Daudel et al. 2007; Schoen et al. 2008). Initial studies have shown that a 3-h incubation of *L. lactis* MG1363 strain, harboring plasmid DNA, with CaCo-2 cells, results in the transfer of the plasmid into the eukaryotic cells and expression of the plasmid (Guimaraes et al. 2006). DNA transfer has also been observed in vivo in mice, after oral administration of the *L. lactis* strain producing the tested allergen (BLG protein—cow β-lactoglobulin). The presence of complementary DNA (cDNA) and protein production in the cells of the small intestine, and the production of specific IgG and IgA antibodies, were both reported. The entry mechanism for the *blg* gene remains unexplained. There are at least two possibilities: either the eukaryotic cells take up plasmid DNA that is released into the intestine from *L. lactis*, or *L. lactis* has been taken up by the eukaryotic cells (Chatel et al. 2008).

Because LAB are generally thought to be incapable of invading eukaryotic cells, strains were designed to interact with eukaryotic cells, which would lead to their internalization. Plasmid DNA transfer into eukaryotic cells has been intensively studied using *L. lactis* that either extracellularly expresses FnBPA protein (a fibronectin binding protein A of *S. aureus*) or InlA (*L. monocytogenes* internalin). The above-described *blg* gene and the *gfp* gene (encoding green fluorescence protein) were used as reporter genes (cDNA). FnBPA, tested both in vitro and in vivo, increased the amount of DNA of the reporter gene in eukaryotic cells, but did not increase the quantity of the antigen produced. The data additionally suggested different mechanisms for in vitro and in vivo entry (Pontes et al. 2012). Similarly, elevated levels of invasiveness and delivery of the GFP-coding plasmid were observed in the in vitro experiment using *L. lactis* that expressed the major InlA internalin of *L. monocytogenes*. The receptor for internalin A is E-cadherin. Due to the structure of the various E-cadherins, InlA recognizes human but not murine E-cadherin. The construction of a *L. lactis* NZ9000 strain exposed the mutated form of InlA, which binds to murine E-cadherin, making it possible to carry out in vivo experiments in the murine model. These, like the in vitro experiments, showed increased invasiveness of the strain, though production of the analyzed protein (BLG) was not increased in vivo (Innocentin et al. 2009; de Azevedo et al. 2012). The in vitro analyses above, as well as the data on immunization against *S. pneumonia* with pure plasmid DNA, obtained from in vivo experiments (Ferreira et al. 2006; Vadesilho et al. 2012), suggest a high potential for application the LAB as DNA vaccines.

## Modulation of the activity of the immune system

Cytokines play an important role in cell signaling, transmitting stimuli and directing immune responses. Attempts are being made to use LAB strains to carry particles that can control the type of induced immune response, with the main focus on their use as therapy for inflammatory diseases of the gastrointestinal tract (Wells and Mercenier 2008). LAB vectors that express antigens of pathogenic microorganisms or secrete cytokines into the environment should evoke a more effective immune response. The first studies on using cytokines in immunization with *L. lactis* involved intranasal administration in mice of an *L. lactis* strain, producing both the TTFC and biologically active murine cytokines IL-2 or IL-6. Coproduction induced significantly higher levels of specific IgG antibodies than when *L. lactis* produced only TTFC (Steidler et al. 1995, 1998a). The use of *L. lactis* expressing chicken interleukin 2 (chIL-2) together with avian influenza hemagglutinin (H5) also revealed adjuvant properties of chIL-2, and the study suggests that *L. lactis* could be a promising candidate as antigen carrier in vaccines against avian flu (Szatraj et al. 2014). In recent years, the influence of IL-12 on the immune system has been extensively studied. One of the promising examples of IL-12 in immunoprophylaxis is the development of a vaccine against leishmaniasis, an infectious disease caused by *Leishmania* parasites that kills 50,000 people a year worldwide. Two *L. lactis* strains were developed, one producing an *L. major* antigen, LACK, anchored to the bacterial cell wall, and the second secreting active murine IL-12. Subcutaneous administration of both strains to mice resulted in a specific Th1 response (Hugentobler et al. 2012b). A strain capable of simultaneous expression of the LACK antigen and IL-12 evoked an immune response that protected mice from a subsequent infection by *L. major* (Hugentobler et al. 2012a).

## Summary

In the two decades since the first reports, many studies of LAB as vaccine vectors have been published. Most of these used LAB as carriers of foreign antigens to immunize against pathogens that enter the human body through the mucous membranes of the respiratory and digestive tracts. LAB strains show enormous potential, especially the use of members of the *Lactobacillus* genus for immunoprophylaxis. However, results of the many studies are difficult to compare and interpret due to the high diversity of bacterial physiology and genetics within the LAB group. The LAB are characterized not only by a varied ability to survive in the intestinal environment but also by a varied interaction of the bacteria with epithelial surfaces and lymphatic cells, even among strains of the same species. In conclusion, it should be emphasized that each potential vaccine that uses LAB as antigen carriers will probably require different genetic constructs, in terms of the localization of the antigen in the carrier cell, and different immunization schedules. Furthermore, the efficacy of these constructs will only be known through in vitro and in vivo testing. GEM particles are nonlive and nonrecombinant derivative of *L. lactis*. Thus, this strategy eliminates all risks attributable to GMO application. However, using GEMs will require complicated immunization procedure. They are not effective when used for oral immunization but efficacious when administered by intranasal route. On the other hand, so far tested genetically modified live LAB carriers of heterologous antigens contain plasmids harboring genes conditioning antibiotics resistance. Thus, introducing these strains for human or animal vaccination will require construction of specific host–vector balance systems in which the chromosomal deletions of housekeeping genes will be complemented by wild-type copies of genes present on plasmids. This strategy, which eliminates drug-resistance markers in live vaccines, has been successfully used in case of other live vaccines. Based on so far published data, using GEM vs live LAB strains for human immunization will be determined by the required type of immune response as well as by the most efficient route of immunization, and it has to be verified in clinical trials.
